# Pharmacokinetic and pharmacodynamic responses in adult patients with Chagas disease treated with a new formulation of benznidazole

**DOI:** 10.1590/0074-02760150401

**Published:** 2016-03

**Authors:** Marisa Liliana Fernández, Maria Elena Marson, Juan Carlos Ramirez, Guido Mastrantonio, Alejandro Gabriel Schijman, Jaime Altcheh, Adelina Rosa Riarte, Facundo García Bournissen

**Affiliations:** 1 Instituto Nacional de Parasitología Dr M Fatala Chabén, Buenos Aires, Argentina; 2 Universidad Nacional de La Plata, Facultad de Ciencias Exactas, Departamento de Ciencias Biológicas, Área de Toxicología, La Plata, Argentina; 3 Instituto de Investigaciones en Ingeniería Genética y Biología Molecular, Laboratorio de Biología Molecular de la Enfermedad de Chagas, Buenos Aires, Argentina; 4 Hospital de Niños Ricardo Gutiérrez, Servicio de Parasitología-Chagas, Buenos Aires, Argentina

**Keywords:** pharmacology, Chagas disease, benznidazole

## Abstract

Pharmacological treatment of Chagas disease with benznidazole (BNZ) is effective in children in all stages, but it is controversial in chronically infected adults. We report the pharmacokinetics and pharmacodynamics in six adult patients with Chagas disease treated with the new BNZ formulation (ABARAX^®^) in doses between 2.5-5.5 mg/Kg/day. All but one patient had plasmatic BNZ concentrations within the expected range. All patients finalised treatment with nondetectable *Trypanosoma cruzi*quantitative polymerase chain reaction, which remained nondetectable at the six month follow-up. Our data suggests parasitological responses with the new BNZ and supports the hypothesis that treatment protocols with lower BNZ doses may be effective.

Chagas disease, a zoonotic disease affecting six million people in Latin America, is caused by the haemoflagellate protozoan known as *Trypanosoma cruzi*(WHO 2015). Treatment for Chagas disease, developed more than 40 years ago, is highly effective during the acute phase of the infection and in chronically infected children (de Andrade et al. 1996, Sosa-Estani et al. 1998), but its efficacy in the chronic stage in adults is still subject of intense controversy (Villar et al. 2014). However, trypanocidal treatment for chronic Chagas disease has increased in the past decade due to recommendations by multiple national and international organizations.

Benznidazole (BNZ) is currently considered the trypanocidal drug of choice for the treatment of Chagas disease, and nifurtimox a valid alternative option. Limited human data is available for both drugs, leading to a limited understanding of their clinical pharmacology, particularly of the mechanisms involved in the therapeutic response and the high rate of adverse drug reactions (ADRs) (Castro et al. 2006, Hasslocher-Moreno et al. 2012).

We studied six adult patients with Chagas disease treated with the new BNZ formulation, ABARAX^®^. Chagas disease was diagnosed by means of at least two positive serological tests for *T. cruzi* (indirect immunofluorescence, enzyme immunoassay and/or indirect haemagglutination). None of the patients had received trypanocidal treatment before. All patients presented laboratory tests within normal values and a negative pregnancy test for women in reproductive age. During treatment, patients were advised to follow a low fat diet, excluding histaminergic foods and alcohol. Contraception was also indicated for women in reproductive age. Clinical laboratory tests (haemogram, liver enzymes, and renal function tests) were obtained at 25 and 45 ± 5 days of treatment; medical follow-up visits were planned at 35 ± 7 days. Real-time PCR for satellite and kinetoplastid DNAs of *T. cruzi* (Ramírez et al. 2015) were done before treatment, at the end of treatment, and after six months of follow-up as an early surrogate biomarker of potential for treatment failure (Molina et al. 2014, Morillo et al. 2015, Pinazo et al. 2015). This study was approved by Bioethics Committee of Fatala Chaben Institute on 1 August 2013; our ethics review board does not provide protocol.

Treatment was scheduled according to current adult treatment guidelines (MSAL 2012). Blood extractions for therapeutic drug monitoring were done under express patient consent, but considered part of required therapeutic measures. Plasma samples were obtained pre and post-doses, for therapeutic drug monitoring purposes, during steady state phase of the drug (i.e., at least after 48 h or after four half-lives from the start of the treatment). BNZ was measured in plasma using a previously published high-performance liquid chromatography method (Marson et al. 2013). All patients consented to the procedures and treatment.

BNZ doses ranged from 4.12-5.50 mg/Kg/day in four patients and 2.50-2.60 mg/Kg/day in two patients (patients 1 and 2) who did not follow medical indications and took half the prescribed dose (i.e, 100 mg bid instead of 200 mg bid) (Table I).

**TABLE I:** Clinical features of treated patients

Patient ID	Age (years)/gender	Place of birth	BNZ dose (mg/kg/day)	BNZ treatment (days)	SatDNA and kDNA qPCRs	Adverse drug reactions
1	45/M	Paraguay	2.60	60	ND/ND/ND	10th day: mild dermatitis 53rd day: mild myalgia in lower limbs
2	33/F	Argentina	2.50	60	NQ/ND/ND	45th day: pruritus 24th day after EOT: cervical lymph node pain
3	33/M	Argentina	5.48	23	ND/-/ND	13th day: mild dermatitis 23rd day: oral mucositis, dysgeusia, and paresthesia in lower limbs
4	29/F	Bolivia	4.12	32	ND/ND/-	9th day: pruritus 26th day: liver enzymes 20 times UNL, hypereosinophilia
5	21/M	Bolivia	4.55	60	NQ/ND/ND	Not observed
6	31/M	Bolivia	4.55	60	NQ/ND/-	Not observed

BNZ: benznidazole; EOT: end of treatment; F: female; M: male; ND: nondetectable; NQ: nonquantifiable (time of measurement: baseline/end of treatment/6 month of follow-up); qPCR: quantitative polymerase chain reaction; UNL: upper normal level.

Measured plasma BNZ concentrations ranged from 4.6-15.0 mg/L (Table II). Ratio of plasma BNZ concentration (mg/L)/BNZ administered dose (mg/Kg/day) showed a narrow range of values, from 1.0-3.0 mg/L of BNZ per mg of drug administered, except for patient 1 (Figure,Table II).

**Figure:**
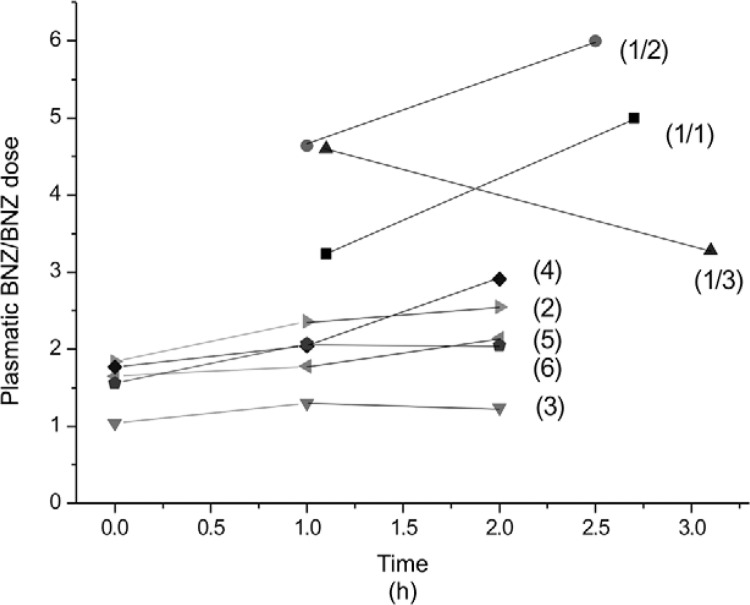
Ratio of plasma benznidazole (BNZ) concentration (mg/L)/BNZ administered dose (mg/Kg/d) during steady state phase. Patient 1 had two samples per day from three different days showed a high ratio of BNZ concentration all the times (1/1: patient 1, samples day 1; 1/2: patient 1, samples day 2; 1/3: patient 1, samples day 3). Patient 2 took half the indicated dose, her plasma drug concentration was normalised according to BNZ administered dose, and presented similar ratio as the rest of the patients of the series (patients 3, 4, 5, and 6).

**TABLE II:** Pharmacometric parameters of patients

Patient ID	Time in BNZ treatment (day)	Plasma samples (times per patient)	Sampling time after last intake (hours)	Plasmatic BNZ (mg/L)	Plasmatic BNZ (mg/L)/ BNZ daily dose (mg/Kg)
1^*a,b*^	53	1/1	1.1	8.1 ± 0.1	3.12
		1/2	2.7	12.5 ± 0.6	4.81
	55	2/1	1.0	11.6 ± 0.3	4.46
		2/2	2.5	15.0 ± 0.2	5.77
	60	3/1	1.1	11.5 ± 0.1	4.42
		3/2	3.1	8.2 ± 0.1	3.15
2^*a*^	60	0	0.0	4.6 ± 0.1	1.84
		1	1.0	4.9 ± 0.1	1.96
		2	2.0	5.1 ± 0.1	2.04
3^*c*^	5	0	0.0	5.7 ± 0.5	1.04
		1	1.0	7.1 ± 0.1	1.30
		2	2.0	6.8 ± 0.1	1.24
4^*c*^	5	0	0.0	7.3 ± 0.1	1.77
		1	1.0	8.4 ± 0.1	2.04
		2	2.0	12.0 ± 0.3	2.91
5	17	0	0.0	7.5 ± 0.1	1.65
		1	1.0	8.1 ± 0.1	1.78
		2	2.0	9.7 ± 0.3	2.13
6	5	0	0.0	7.1 ± 0.1	1.56
		1	1.0	9.4 ± 0.1	2.07
		2	2.0	9.3 ± 0.1	2.04

*a*: patients 1 and 2 - 2.5 and 2.6 mg/kg/day dose;*b*: patient 1 had two samples per day from three different days; *c*: patients 3 and 4 - treatment was interrupted; BNZ: benznidazole.

Patient 1, who took half the prescribed dose (i.e., 2.5 mg/Kg/day BID), had BNZ plasma levels higher than those patients taking full dose. This patient was not taking any other medications, nor had any diseases or conditions known to affect BNZ pharmacokinetics. Actual dosage was confirmed by pill counts (number of BNZ tablets in the pill bottle was compatible with the patient taking a reduced dose, i.e, twice the number of tablets expected were observed in the bottle) and intensive reviewing of drug intake history with the patient.

Patient 2, who also took half the prescribed BNZ dose, had BNZ concentrations compatible with this dosage (Table II). In spite of lower BNZ plasma levels, this patient showed an appropriate parasitic response to BNZ [i.e., nondetectable quantitative polymerase chain reaction (qPCR) for parasite DNA at six months follow-up].

In addition, three out of six patients [patients 2, 5, and 6 (Table I)] had positive qPCRs for *T. cruzi* SatDNA and kDNA at baseline, whereas all patients in this series had nondetectable qPCR at the end of treatment and at six months of follow-up for both qPCR methods. Four patients developed ADRs during treatment and two patients had severe ADRs which required treatment discontinuation (patients 3 and 4). A relationship between plasmatic BNZ dosage and ADRs was not observed in this small number of cases.

Historically, medication availability has been one of the most important health issues related to neglected diseases such as Chagas disease. BNZ was developed and produced by Roche until 2011 when production was discontinued (Jannin & Villa 2007). BNZ production was later taken up by the Brazilian pharmaceutical company LAFEPE and Argentinian laboratory ELEA. The latter developed a new BNZ formulation named ABARAX^®^, which was fast-tracked by the Argentine National Food, Drug, and Medical Technology Agency due to the urgent problem of re-establishing an adequate BNZ supply (Navarro et al. 2012). Unfortunately, emergency approval of the new formulation and the fact that there is no stock of the original drug formulation produced by Roche meant that no bioequivalence studies were carried out (ANMAT 2012). This situation leads to a lack of pharmacological data on drug bioavailability to guide dose adjustments of the new formulation.

We observed no relationship between plasma BNZ values and occurrence of adverse events or their severity in this limited small series. This observation is in line with previous similar studies (Pinazo et al. 2013, Salvador et al. 2015). However, a remarkably high rate of ADRs was observed in our patients group. All patients studied had nondetectable*T. cruzi* SatDNA and kDNA qPCRs at the end of treatment and at six months follow-up, including those who could not complete treatment due to ADRs and those who had lower BNZ plasma levels. This observation shows probably parasitological responses to BNZ treatment even at lower exposures and provides further support to the hypothesis that treatment protocols with lower BNZ doses could be effective, as suggested by previous observations in children (Altcheh et al. 2011) and adults treated with different BNZ formulations. Moreover*in silico* models, computer simulations of biological models show that a lower dose (2.5 mg/kg/d) would be enough to achieve minimum plasma concentrations within the proposed therapeutic BNZ range between 3-6 mg/L (Soy et al. 2015). Lower doses may have, at least in theory, the advantage of a lower incidence of ADRs, the primary cause of discontinuation of drug treatment.

We have no clear explanation for the observed pharmacokinetic behaviour in the patient who disclosed taking half BNZ dose but had BNZ plasma levels higher than patients treated with a full BNZ dose. Unfortunately, little is known about what factors that may affect BNZ pharmacokinetics. Considering that the patient’s dosage was compatible with the number of tablets left in his pill bottle and that he was not receiving any other medications nor had any clinical conditions that could affect BNZ elimination, we may speculate that it is possible that this patient had a polymorphism, hitherto unknown, in the molecular mechanisms involved in the elimination of BNZ that could have led to accumulation to higher concentrations than expected. This is a tantalising possibility that we are currently exploring, but research in this area is significantly hampered by the lack of knowledge on absorption mechanisms, metabolism, and elimination pathways of BNZ up to date.

This study suggests that BNZ responses continue to be high with the currently available BNZ formulation (ABARAX^®^) and provides new support to the hypothesis that treatment protocols with lower BNZ doses may still be effective. Preliminary observations in one of our patients suggest polymorphic behaviour in the elimination of the drug, which needs to be further explored in clinical and basic studies.
